# Recreational Diving Practice for Stress Management: An Exploratory Trial

**DOI:** 10.3389/fpsyg.2017.02193

**Published:** 2017-12-18

**Authors:** Frédéric Beneton, Guillaume Michoud, Mathieu Coulange, Nicolas Laine, Céline Ramdani, Marc Borgnetta, Patricia Breton, Regis Guieu, J. C. Rostain, Marion Trousselard

**Affiliations:** ^1^Laboratoire UMR - MD2, Faculté de Médecine Nord, Aix-Marseille Université, Marseille, France; ^2^Department of Emergency, Ste Anne's Military Hospital, Toulon, France; ^3^Ecole du Val de Grâce, Paris, France; ^4^Pole Réanimation Urgence SAMU Hyperbarie, CHU Ste Marguerite, Marseille, France; ^5^Département de Neurosciences et Contraintes Opérationnelles, Brétigny sur Orge, France; ^6^Institut Nationale de Plongée Professionnelle, Marseille, France; ^7^Union Nationale des Centres Sportifs de Plein Air, Paris, France; ^8^Laboratory of Biochemistry, Timone University Hospital, UMR MD2, Aix Marseille University, Marseille, France; ^9^APEMAC-EPSam EA4360 UDL UFR SHS, Metz, France; ^10^Chaire de Mindfulness, Bien-être au Travail et Paix Économique, Grenoble Ecole de Management, Grenoble, France

**Keywords:** perceived stress, recreational diving, sport, stress, mindfulness

## Abstract

**Background:** Within the components of Scuba diving there are similarities with meditation and mindfulness techniques by training divers to be in a state of open monitoring associated with slow and ample breathing. Perceived stress is known to be diminished during meditation practice. This study evaluates the benefits of scuba diving on perceived stress and mindful functioning.

**Method:** A recreational diving group (RDG; *n* = 37) was compared with a multisport control group (MCG; *n* = 30) on perceived stress, mood, well-being and mindfulness by answering auto-questionnaires before and after a 1-week long UCPA course. For the diving group, stability of the effects was evaluated 1 month later using similar auto-questionnaires.

**Results:** Perceived stress did not decrease after the course for the MCG [ The divers showed a significant reduction on the perceived stress score (*p* < 0.05) with a sustainable effect (*p* = 0.01)]. An improvement in mood scale was observed in both groups. This was associated to an increase in mindfulness abilities.

**Conclusions:** The practice of a recreational sport improves the mood of subjects reporting the thymic benefits of a physical activity performed during a vacation period. The health benefits of recreational diving appear to be greater than the practice of other sports in reducing stress and improving well-being.

## Introduction

Chronic stress is a common complaint for middle-aged populations. Stress can be defined as a pattern of cognitive appraisal, physiological responses and behavioral tendencies that occur in response to a perceived imbalance between situational demands and the resources needed to cope with them. Chronic stress can occur in response to everyday stressors that are ignored or poorly managed. The reaction of individuals to chronic stress is theorized in the general alarm syndrome (Selye, 1956) and allostasis theories (McEwen, 2004), contributing to high biological cost featuring the allostatic load (Chrousos, 2009). Excessive chronic stress, which is constant and persists over an extended period of time, can be psychologically and physically debilitating. The consequences of chronic stress are serious. It constitutes a public health problem by increasing morbidity and mortality (Sayers, 2001). It notably contributes to anxiety and depression, which increase the risk of heart disease (Anderson and Anderson, 2003). In the professional sphere, it impacts job performances (Scullen et al., 2000). For the individual, it induces emotional and mood disturbance (Głebocka, 2016) and decreases well-being and quality of life (Palgi, 2013). Whether any biological marker for chronic stress is validated, robust questionnaires are available for assessing the level of perceived stress of subjects. Namely, the Cohen perceived stress scale (PSS; Cohen et al., 1983) allows a linear measurement of perceived stress, with the threshold found to be related to mental disorders (Collange et al., 2013).

It is well-known that each individual reacts in a specific way when they are exposed to a stressful situation. The individual's alteration depends on many variables, particularly when studied in the psychology of health (Bruchon-Schweitzer, 2002) and positive psychology (Martin-Krumm and Tarquino, 2011). The described concepts take into account how the subject's history and personality contribute to the assessment of the situation and what the subject will do (social support, stress and control of the perceived situation), the regulation by cognitive adjustment (*coping* centered on the task) or emotions (defuse the situation) that the subject will feel and the type of response that they subsequently implement (Bruchon-Schweitzer, 2002).

Within this frame of reference mindfulness must be taken into account. Mindfulness is a state of consciousness resulting from intentionally focusing one's attention on the present moment, without judging the experience that unfolds moment after moment (Kabat-Zinn, 2003). It is considered as one of the mind's natural resources, present in all individuals to varying degrees. The *Mindful* subject is a subject who chooses to consciously receive what is happening to his/her conscience with an attitude of openness, receptivity and non-judgment, allowing him- or herself not to be imprisoned by negative effects.

To deal with chronic stress and to improve stress management is challenging. Psychological fitness aimed at regulating emotions and mood disorders to enable improved effectiveness under stress and faster recovery from psychological stress (Bates et al., 2010; Mullen, 2010). Many programs exist that are centered on stress and emotional regulation by using breathing and relaxation exercises. Particularly meditation exercises allow mindfulness functioning to develop and improve the abilities for emotion regulation and for coping with stress (Chiesa and Serretti, 2009). One of the main concerns for such training is the need for the subject's regular compliance for the practice. Daily compliance is key for training efficacy.

Practicing physical activity, aerobic or anaerobic, has also been proposed for a long time as an ecological method to reduce stress and to improve emotion regulation (Paluska and Schwenk, 2000). Moreover, physical activity has an indisputable interest in the prevention and treatment of mental disorders associated with anxiety (Fox, 1999; Penedo and Dahn, 2005) and beyond that, there are somatic benefits, notably for cardiovascular diseases (Paffenbarger et al., 1986; Penedo and Dahn, 2005). These benefits are all the more important when the practice is regular (Petruzzello et al., 1991): The practice of moderate activity (at least 3 h per week) or intense activity (at least 20 min three times a week).

Scuba diving as a recreational sport has seen a tremendous rise ever since the diving structures have become more organized and offer access to reliable equipment. Every year, more than 260,000 people dive as part of the French Federation of Studies and Aquatics Sports. Most of the studies in this particular field tend to focus on the dangers and incidents of the sport, but not many actually cover the benefits of scuba diving on health in general and on stress management in particular. Yet immediately after diving, many divers experience a salutogenesis effect from the sport described as a state of well-being. Theoretically, from a psychological point of view, the analysis of scuba diving suggests that it favors experiencing a state of full consciousness and of openness associated with slow and ample breathing. Moreover, the homogenous stimulation of somaesthetic and proprioceptive captors during diving could improve senses perception, movement and body sensations. These psychological characteristics are close to those developed during meditation, suggesting that a scuba diving exercise could be considered as a meditation exercise inducing a state of mindfulness and that the repetition of diving should develop mindfulness functioning for regular divers.

Altogether, these elements suggest that the mechanisms of salutogenesis created by meditation in a broader aspect, could indeed be transcribed into scuba diving. Given how beneficial mediation is toward stress management, one could argue that scuba diving, even at a recreational level, could improve any diver's mental health by its virtue in stress management.

The final aim of the study is to investigate the effect of a week's scuba diving training on the level of perceived stress by comparing it with the effect of doing another sports training for a week. The effect on perceived stress level is also examined in terms of developing mindfulness skills. It is also hypothesized that the benefits of a week's scuba diving course on the level of perceived stress lasts 1 month.

## Materials and method

### Subjects

Two middle-class populations registered at the same leisure sports club were included at the same period of the year: one population sample registered for a 1-week recreational scuba diving course (37 subjects, diving group) and one population sample registered for a 1-week multisport course (30 subjects, control group).

Potential participants were excluded from the samples if (i) they were undergoing medical treatment for psychological issues at the time of the study or (ii) they had taken part in a stress management program prior to recruitment for the current study.

### Recruitment and data collection

Subjects received a cover letter supported by the leisure sports club's respective board, inviting participation in the study and stating the exclusion criteria. Medical staff assisted the subjects' recruitment.

The study was conducted in accordance with all applicable regulatory requirements, including the 1996 version of the Declaration of Helsinki and approved by the French Health Service's ethics committee. All volunteers provided written informed consent before participation.

### Protocol

We conducted an open non-controlled and non-randomized interventional study. The exploratory protocol included two similar sessions of standardized auto-questionnaires for both groups (baseline and post-training sessions). A third session 1 month after the training program was added for the diving group only (post 1-month training session) using the same questionnaires.

At each session, subjects fulfilled auto-questionnaires in a “paper and pencil” booklet. They were asked to respond to socio-demographic questions at the first session only. For divers only, questions were added at session one about usual diving practice and at the post-1 month training session about life events that occurred during the month that followed the end of the course.

For each subject, the intervention was a training course lasting 1 week. It included 6 days of sports practice. For divers, diving was carried out using air at a maximum depth of 40-meters, with a maximum of two dives a day. For the multisport group (the non-divers), the course included kayaking, mountain climbing or hiking with a maximum of two activities a day.

### Measures

Primary outcome measure: The Perceived Stress Scale (PSS; Cohen et al., 1983) is a 14-item scale designed to assess subjects' appraisal of how stressful their life situation feels to them. Each self-descriptive statement was evaluated using a five-point Likert scale ranging from 1 (strongly disagree) to 5 (strongly agree). The PSS is recommended for assessing non-specific appraisal because it is found to predict better stress-related psychological symptoms and physical symptoms compared with commonly used life event scales. The most appropriate stress-threshold would be a score strictly superior to 27 for anxiety and 28 for depression (Collange et al., 2013).

Four auto-questionnaires evaluated psychological states as secondary outcome measures.

The Freiburg Mindfulness Inventory-14 is a short form with 14 items developed for people without any background knowledge in mindfulness (Walach et al., 2006; Trousselard et al., 2010). It constitutes a consistent and reliable scale evaluating the state of mindfulness and two subfactors (Kohls et al., 2009): acceptance as an ability to embrace unwanted thoughts and feelings as an alternative to experiential avoidance and being present, which characterizes being in non-judgmental contact with environmental events as they occur. Each self-descriptive statement was evaluated using a four-point Likert scale ranging from 1 (strongly disagree) to 4 (strongly agree).

The Body Connection Scale (BCS, Price and Thompson, 2007) is a 20-item scale designed to assess body awareness with two-faceted sensory awareness and bodily dissociation. Sensory awareness evaluated the ability to identify and experience inner sensations of the body and the overall emotional/physiologic state of the body such as bodily changes/responses to emotions and/or environment (12 items). The concept of bodily dissociation is characterized by avoidance of internal experience. Bodily dissociation has experiential aspects including normal everyday experiences, such as distraction from bodily experience or emotional disconnection (8 items). Each self-descriptive statement was evaluated using a four-point Likert scale ranging from 0 (strongly disagree) to 3 (strongly agree).

Warwick-Edinburgh Mental Well-Being Scale (WEMWBS; Tennant et al., 2007; Trousselard et al., 2016) covers both affective constructs, including the experience of happiness and constructs representing psychological functioning and self-realization (Keyes, 2007). WEMWBS comprises 14 items relating to the previous week, with responses that range from (1) “none of the time” to (5) “all of the time.”

Mood was evaluated using the abbreviated version of the Profile of Mood States (POMS; Shacham, 1983). It consisted of an adjective checklist of 37 items that range from (0) “not at all” to (4) “extremely.” The subjects were asked to answer according to their present mood. Six factors were then calculated: anxiety-tension, depression-dejection, anger-hostility, fatigue-inertia, vigor-activity and confusion-bewilderment.

### Statistical analysis

All data, expressed as mean (SD), were treated as ordinal data except for gender and marital status.

For each psychological variable, the distribution normality was tested as the variance homogeneity was also tested (Levene Test). In general, the normality and homogeneity conditions were respected (exceptions will be reported case-by-case). For every questionnaire, the internal coherence was tested using the Cronbach Test (Cronbach and Meehl, 1955). It appeared that each questionnaire showed an acceptable psychometric quality (Cronbach alpha ≥0.7).

The effect of the sport's training was carried out separately using student tests on primary and secondary outcomes. For the 1-month persistence on diving effects, analyses of variance (ANOVA) with a time session (three sessions) were performed as within subjects' effect. This was done separately for the primary outcome and each secondary variable. For significant effects, *post-hoc* analyses using Newman-Keuls were applied. When data are expressed as categorical or percentages, Chi-square tests were used.

In some very specific cases (non-obvious distribution normality, non-homogeneity of variances), non-parametric tests were used. This is particularly true for the POMS questionnaire and its subfactors. We used the Wilcoxon test for paired samples, the Mann-Withney tests for independent samples and Friedman's ANOVA test for multiple paired samples.

All analyses were performed with SPSS 17.0 for Windows (SPSS GmbH Software, Munich). We judged *p* < 0.05 as significant. When *p* < 0.1, results were expressed as a tendency to a difference.

## Results

### Participants

Table 1 describes socio-demographic characteristics according to the sample population.

**Table 1:** Comparisons of the two samples population.

		**Diver group M (*SD*)**	**Control group M (*SD*)**	***P*-value (*X*^2^ or *t*)**
Sample size		37	30	
Age	Years	41.83;10.81	29.53;5.28	*<0.001*
	[Range]	[30–52]	[24–35]	
Gender	Male (%)	29 (78.37%)	8 (21.63%)	*<0.001*
	Female (%)	8 (21.63%)	23 (76%)	
Marital status	Couple (%)	23 (62.16%)	5 (16.66%)	*<0.001*
	Single (%)	14 (37.83%)	25 (83.33%)	
Educational level	Undergraduate studies (%)	3 (8.1%)	1(3.33%)	0.11
	Graduate studies (%)	34 (91.9%)	29 (96.67%)	
Perceived stress		35.1 (8)	36.8 (5.2)	0.31
POMS	Tension anxiety	5.6 (4.4)	4.4 (4.5)	0.27
	Depression	2 (3.3)	3.4 (5.5)	0.21
	Anger	3.5 (4.1)	2.6 (5.3)	0.39
	Confusion	2.9 (3.2)	3.6 (3.5)	0.36
	Fatigue	4.2 (4.1)	4.9 (4)	0.5
	Activity Vigor	13.2 (4.6)	11.1 (3.5)	*0.04*
	Negative Mood	18.4 (15.5)	19 (20.6)	0.88
Mindfulness	Total	38.4 (7)	35.9 (3.8)	0.08
	Presence	17.7 (3.3)	16.8 (2)	0.19
	Acceptation	20.5 (4.1)	19 (3.1)	0.11
Body connection	Conscience	20.9 (5.1)	23.6 (5.6)	*0.04*
	Dissociation	14.6 (6)	14.7 (3.4)	0.9
WEMWBS		50.7 (8.4)	47.8 (7.2)	0.14

*M, mean; SD, Standard Deviation; X^2^, Chi-square test; t, student test*.

*Italic p values indicated significant differences*.

Concerning this diving sample population, 59.9% of participants have a level 2 diving level at best, 55% mentioned diving as a hobby; 64.9% have dived at least 20 times in the last 12 months and 16.2% dived more than 60 times in that timeframe. During the diving course, divers all dived with air at various depths, some of which might induce narcosis (when deeper than 30 meters). Fifty percent of the participants reported being to a depth where narcosis occurs.

As shown in Table 1, there is no notable difference for psychological profile at baseline session between the two groups: diver group exhibited a higher level of Activity Vigor at the POMS and a lower level of body conscience at the BCS-subscale. There is no significant difference between men and women, among young or older people (above the 33 years-old median) on the psychological scores.

83.78% of the subjects in the diver group exhibited scores above the clinical threshold of 27 and were considered stressed at baseline. 96.67% of the subjects in the control group were considered stressed at baseline; there is no difference for the number of subjects above the clinical threshold between the two groups (*X*^2^ = 2.94, *p* > 0.05).

### Sports practice effect on the primary outcome

The perceived stress significantly dropped after the recreational diving course (*t*-test, *p* < 0.01) whereas no significant change was observed after the multisport course (*t*-test, *p* = 0.24).

We observed a course-induced effect of the level of perceived stress (*F* = 7.46; *p* = 0.02). The drop in the perceived stress score is not different between groups (divers vs. control group: *F* = 0.004; *p* = 0.95). There is no interaction between the course-induced effect and the group (*F* = 1.36; *p* = 0.25).

Five subjects in the group of divers reduced their perceived stress level lower than the clinical threshold upon course completion, whereas only two subjects in the control group did (*X*^2^ = 3.91; *p* = 0.048). The number of subjects decreasing their score under the clinical threshold was higher after the diving course (13.51%) than after the multisport courses (6.67%) (*X*^2^ = 3.91, *p* = 0.04). The Number Needed to Treat (NNT) with diving to see a one-person benefit (under the threshold) is 8 against 17.

### Sport practice effects on secondary outcomes

Table 2 compares the effects of the two sports courses for the mood subfactors and for the well-being. For the scores concerning depression, tension-anxiety, anger and confusion, a significant decrease was observed after the course for the two groups. Fatigue mood level only decreased for the control group (*t* = 2.19, *p* = 0.03). Activity-vigor mood level only increased for the control group after the course (*t* = −2.37, *p* = 0.02). Well-being scores significantly increase for both groups.

**Table 2:** Comparisons for each sample between before and after the course.

	**Diving group**		**Control group**	
	**M (*SD*)**	***p***	**M (*SD*)**	***p***
Tension anxiety	2,14 (2.97)	*<0.01*	1.43 (2.38)	*<0.01*
Depression	1,06 (2.11)	*0.03*	1.5 (2.69)	*0.03*
Anger	1,17 (1.44)	*<0.01*	0.63 (1.27)	*0.04*
Confusion	1,53 (1.99)	*<0.01*	1.9 (2.68)	*<0.01*
Fatigue	4,22 (3.43)	>0.05	3.47 (2.77)	*0.03*
Activity vigor	13,94 (4.86)	>0.05	12.74 (4.51)	*0.02*
Well-being	52.92(8.34)	0.02	51.51(6.48)	*<0.01*

*M, mean; SD, Standard Deviation*.

*Italic p values indicated significant differences*.

Table 3 showed differences on mind-body variables after the course for both groups. The diving course induced an increase in global mindfulness functioning (*t* = −1.97, *p* = 0.05) with an improvement in the acceptation sub factor (*t* = 2.29, *p* = 0.03) associated to a decrease in bodily dissociation (*t* = 1.99, *p* = 0.05). The multisport course induced an increase in global mindfulness functioning (*t* = −2.21, *p* = 0.04) associated to an increase in bodily dissociation (*t* = −1.99, *p* = 0.05).

**Table 3:** Comparisons for each sample between before and after the course.

	**Diving group**		**Control group**	
	**M (*SD*)**	***p***	**M (*SD*)**	***p***
Mindfulness	39.65 (6.57)	*0.05*	37.27 (3.99)	*0.04*
Mindfulness-acceptation	21.62 (3.76)	*0.03*	19.86 (2.9)	0.1
Mindfulness-presence	18.06 (3.49)	0.46	17.41 (2.08)	*0.16*
Bodily conscience	19.51 (6.58)	0.1	24.36 (6.31)	0.33
Bodily dissociation	13.1 (5.03)	*0.0*5	16.57 (4.55)	*<0.01*

*M, mean; SD, Standard Deviation*.

*Italic p values indicated significant differences*.

### Persistence of the diving effects

Half of the divers filled out questionnaires 1 month after the course (48.65%) for this specific subsample and answered all three sessions.

For the primary outcome, a significant session effect was observed with a decrease in the perceived stress score (*F* = 7.48, *p* < 0.01). *Post-hoc* analyses show that the perceived stress score measured at baseline was higher than the scores at the end of the course (*p* = 0.02) and for 1 month afterwards (persistence: *p* < 0.01). No difference is observed between the scores at the end and 1 month after the course (*p* > 0.05).

On secondary outcomes diving effect persistence was also observed. For tension-anxiety score, results showed a significant effect of the sessions (*F* = 7.49, *p* < 0.01). *Post-hoc* analysis showed that the tension-anxiety score measured at baseline was higher than the scores at the end of the course (*p* = 0.02), and tended to remain lowered 1 month afterwards (persistence: *p* = 0.06). For bodily dissociation score, results showed a significant effect of the sessions (*F* = 12.65, *p* < 0.01). *Post-hoc* analysis showed that the bodily dissociation score measured at baseline was higher than the scores at the end of the course (*p* = 0.01) and tended to remain lowered 1 month afterwards (persistence: *p* = 0.09).

## Discussion

This study investigated the psychological effect of a 1-week scuba diving course compared to another 1-week sports course. It showed that this particular sport practice induced a decrease in perceived stress level, which was not observed after a multisport course. This improvement in divers' appraisal of how stressful their life situation feels to them reduced the risk for developing psychological symptoms of 13.51% for divers. In comparison, only 6.67% of the multisport practitioners decreased their score under the clinical threshold of the perceived stress scale. Moreover, such stress management improvement was only associated to an increase in well-being for diving practice. Concerning sport practice effects on mood, both sport courses decreased negative mood in terms of tension-anxiety, depression, anger, and confusion. However, they exhibited opposite effects on fatigue and activity-vigor moods: fatigue and activity-vigor were higher after the diving course whereas they decreased after the multisport course. The reported fatigue after a 1-week diving course appears to be considered as physical as it was not associated with any psychological symptoms that mimic depression or psychological fatigue. In line, the improvement in stress perception after the 1-week diving course appeared as a beneficial effect that lasted for at least a month.

Altogether, such improvements in mental health have already been described in literature (Paluska and Schwenk, 2000; Penedo and Dahn, 2005). However, comparisons between the types of physical activity are scarce. Our results suggest that different patterns of sport practice benefits exist in terms of stress regulation, improving well-being and fatigue. One of the explanations might involve a difference in a stronger mind-body connection depending on the sport activity. Indeed, we observed that the diving course improved mindfulness functioning, acceptance attitude and decreased bodily dissociation whereas the multisport course induced an increase in global mindfulness functioning associated with an increase in bodily dissociation.

Concerning mechanisms that could be involved in the diving benefits, a possible depth effects, implying narcosis, must be proposed although the psychological data recorded in this exploratory study do not allow to go further to confirm this statement. Furthermore, two main psychological mechanisms could be involved in the diving body-connection improvement: specific proprioceptive and somesthetic stimulations and deep breathing induced by the use of the pressure regulator. Body perception involves the processing of sensory information, or sensory integration and refers to this process by which the brain receives a message through the senses and transforms it into an appropriate behavioral response (Miller et al., 2007). It participates in mind-body connection. The motor activity during diving leads to singular sensory pattern by associating proprioceptive and somesthetic stimulations exerted to the entire body. If mind-body connection cannot be reduced to these peripheral proprioceptions, this latter contributes strongly to it. In the strict sense, proprioception defines a coupling system that intervenes in the perception of movement (kinaesthesia) and body positioning (statesthesia). It constitutes a moto-proprioceptive loop leading to proprioceptive sensitivity, which allows sensitivity to the deep organs (bones, joints, muscles, ligaments). Data reinforces the importance of the loop in the genesis of self-perception: (i) there is reaffirming sensory flux specific to each movement that can be defined as a true signature; (ii) there is a continuous solicitation of this system by the form and deformations of the body which would allow the constitution of reliable invariants relative to the body, by convoking the body itself. These elements give proprioception a primacy in the sense of a primary meaning capable of calibrating others, of playing the role of a matrix. Proprioceptive stimulation when diving associating the sensory flows with the movement of the subject and the somesthetic pattern of stimulation may intervene in both the regulation of the postural tone and the bodily experience and thus may increase the perception of the Self (Roll, 2003). On the other hand, the breathing that the subject establishes during a dive is a deep, steady and slow breathing. Such respiration is known to increase heart rate fluctuations and sinus respiratory arrhythmia through a better balance in the autonomous nervous system (Shaffer et al., 2014). This autonomous functioning is associated with the maintenance of a physiologically efficient and highly regenerative inner state. This psychophysiological mode is conducive to healing and rehabilitation, emotional stability and optimum performance (McCraty et al., 1995, 1998). Although, we cannot conclude as to which specific component of diving creates these mind-body connection improvements. The difference observed between the two sport groups could be explained by an optimization of the respiratory and proprioceptive profits induced by diving.

This exploratory study suffers from multiple biases, starting with the population studied. Matching of sociological and demographical data, such as age, gender, education and psychological data could not be set up. Entries for diving practice show that middle-aged men are the most frequent. The opposite is observed for multisport courses which mainly included young females. Second, studying perceived stress and psychological factors can prove to be tricky, as they are variables that evolve on one's personal situation and evolve potentially in a big way during a period when one does not work. The “break effect” thus plays an important role in our results. Though we can still observe that divers are not less tired at the end of their courses, compared with the group control whose tiredness level decreases. This puts this “break effect” into perspective that we would have expected from both groups. Thirdly, the remanence effect is studied in a small group (18 of the 37 divers answered the final test). We can consider that this group may have appreciated the most benefits of this study, therefore answered 1 month after the diving sessions. Finally, the hourly volume of daily physical activities during the leisure club sport courses could not be compared exactly between the diving courses and multisport courses. These would be equivalent given the scheduled week program. Weather conditions during all considered diving courses were good enough for all dives to be completed.

## Conclusion

Diving as a recreational activity offers multiple health benefits, such as a decrease in perceived stress and an improvement of multiple psychological factors associated with mindfulness abilities. There does not seem to be any modification of the perceived stress level in our group control, but psychological factors are also improved. The “holiday's effect” paired with a physical activity, both play a big role in both groups and we can thus confirm the studies that were previously made on the subject.

Well-controlled studies are needed to clarify the mental health benefits of diving; by keeping in mind that scuba diving is a risky activity if safety regulations are not adhered to, even though it has a polarity with meditation. A new diver would not be expected to be familiar with the aquatic environment; the immersion reflex and the use of specific equipment (with an unknown reliability) can be a source of stress. This can lead to decompression accidents, drowning or pulmonary edema. Nevertheless, it is relevant to discuss the potential applications of these preliminary results for preventing anxiety and depressive syndromes or for promoting recovery after a stressful period.

## Author contributions

FB, MC, MB, CR, PB and MT was involved in the conception and trial design. FB, NL, RG, JR, and MT contributed reagents, materials, analysis tools. FB, GM and MT wrote the draft of the article and contributed to the refinement of the study protocol and provided expert insight.

### Conflict of interest statement

The authors declare that the research was conducted in the absence of any commercial or financial relationships that could be construed as a potential conflict of interest.
